# Consumption of sugar-sweetened beverages and type 2 diabetes incidence in Thai adults: results from an 8-year prospective study

**DOI:** 10.1038/nutd.2017.27

**Published:** 2017-06-19

**Authors:** K Papier, C D'Este, C Bain, C Banwell, S Seubsman, A Sleigh, S Jordan

**Affiliations:** 1 National Centre for Epidemiology and Population Health (NCEPH) and Department of Global Health, Research School of Population Health, College of Medicine, Biology and Environment, The Australian National University, Canberra, Australia; 2 Population Health Department, QIMR Berghofer Medical Research Institute, Brisbane, Queensland, Australia; 3 Thai Health-Risk Transition Study, School of Human Ecology, Sukhothai Thammathirat Open University, Nonthaburi, Thailand; 4 The School of Public Health, The University of Queensland, Brisbane Queensland, Australia

## Abstract

**Background:**

The global prevalence of type 2 diabetes mellitus (T2DM) is high and is increasing in countries undergoing rapid socio-economic development, including Thailand. Sugar-sweetened beverage (SSB) intake may contribute to the risk of developing T2DM. However, few studies have assessed this association in Asian populations, and the results have been inconsistent. We aimed to assess that association in a prospective study of Thai adults.

**Methods:**

Data were from Thai Cohort Study participants surveyed in 2005, 2009 and 2013. The nation-wide sample included adult cohort members who were free of diabetes in 2005 and who were followed-up in 2013 (*n*=39 175). We used multivariable logistic regression to assess associations between SSB intake and eight-year T2DM incidence. We used a counterfactual mediation analysis to explore potential mediation of the SSB intake and T2DM-risk relationship.

**Results:**

In women (but not men) consuming SSBs once or more per day (versus rarely) was associated with increased T2DM incidence at the 8-year follow-up (odds ratio (OR)=2.4, 95% confidence interval (CI) 1.5–3.9). Obesity in 2009 was found to mediate ~23% of the total association between SSB intake in 2005 and T2DM risk in 2013 (natural indirect effect 1.15, 95% CI (1.02, 1.31).

**Conclusions:**

Frequent SSB consumption associated with higher T2DM incidence in women but not men. We found that a moderate proportion of the SSB-T2DM relationship was mediated through body mass index (BMI). Our findings suggest that targeting SSB consumption can help prevent a national rise in the incidence of T2DM.

## Introduction

Many low and middle income countries (LMICs) have experienced considerable social and economic development in recent decades inducing a ‘health-risk transition’ characterized by changes in environment, health behaviour and emergence of non-communicable diseases such as type 2 diabetes mellitus (T2DM).^1, 2^ Thailand is one such country that has experienced an increase in T2DM prevalence from 2.3% in1991^(ref. 3)^ to 8.0% in 2015.^2^

Increasing sugar consumption in Thailand may relate to increased T2DM. Between 1983 and 2009 sugar consumption jumped from 12.7  to 31.2 kg per person per year,^4^ much in carbonated soft drinks.^5^ The 2009 National Health Examination Survey (NHES) shows that frequency of approximately daily intake of carbonated soft drinks doubled (from 7.9 to >16%) among Thais aged 15 years or older since 2003.^6^

Sugar-sweetened beverage (SSB) consumption, which includes sweetened carbonated soft drinks, has been linked to increased T2DM risk in African and Caucasian populations,^7, 8, 9^ with some research suggesting the association is mostly mediated by increasing body mass index (BMI).^10, 11^ There are limited and inconsistent data on SSB consumption and T2DM risk among Asian populations.^12, 13, 14^

SSBs are an ideal target for public health interventions to help control the T2DM epidemic since they have no nutritional value, are not rooted in Thai culinary culture, and do not protect against disease.^15^ Furthermore, past performance of the Thai government in banning tobacco promotion suggests that parallel approaches to controlling SSBs would be possible.^16^ The aims of this study were to clarify the association between SSB consumption and T2DM risk over an 8-year period and whether they are mediated by BMI in a prospective study of Thai adults, the Thai Cohort Study.

## Subjects and methods

### Study population

The Thai Cohort Study (TCS) is a prospective study of 87 151 Thai adults enrolled at Sukothai Thammithirat Open University (STOU), established to examine the ‘health-risk transition’ in Thailand.^17^ In 2005 all 200 000 students enrolled at STOU were mailed a questionnaire covering sociodemographic, health and lifestyle factors, and health outcomes (including diabetes). Overall 87 151 (44%) students returned the completed questionnaires forming the baseline cohort. Follow-up questionnaires were sent in 2009 and 2013 and respectively 60 569 (70% response rate) and 42 785 (71% of 2009 participants) were returned.

#### Eligibility

Participants were eligible for this study if they reported that they did not have diabetes at baseline, had a valid SSB intake response in 2005, and provided a diabetes status in 2009 and/or 2013.

#### Assessment of T2DM status

Participants were classified as having diabetes if they responded positively to the question ‘Have you ever received a confirmed diagnosis from a doctor that you definitely have diabetes?’ by 2013. A validation study of self-reported diabetes conducted among TCS participants indicated that the accuracy of diabetes self-report was high (82%).^18^

#### Assessment of SSB intake

In each questionnaire participants were asked about their SSB consumption. In Thai, this translated to any carbonated sweetened beverage or soda and did not distinguish between regular and diet soda intakes. However, for consistency with previous TCS work^19^ and the literature, we use the term SSBs throughout. SSB consumption at baseline was reported in categories and classified as: <1 weekly, 1 to 6 per week, or ⩾1 per day.

#### Assessment of covariates

Questionnaire items included sociodemographic characteristics: age, income, education level, and area of residence (urban/rural); lifestyle factors: smoking (never smoked, ex-smoker and current smoker) and alcohol consumption (never, ex-drinker, occasional/social drinker and regular drinker); fruit and vegetable consumption(categorized as <2 or ⩾2 serves/per day), and consumption of deep-fried food (<3 × per month, 1–6 per week, 1+ per day). Leisure physical activity, reported as number of sessions per week of strenuous, moderate or mild exercise, was weighted (‘2 × strenuous+moderate+mild+walking’ exercise sessions)^20^ and categorized by sessions per week (none, 1–7, 8–14, 15 or more).^21^ Participants also reported height and weight. Body mass index (BMI—weight in kg divided by height in m^2^) was categorized as recommended for Asian populations.^22^ The questionnaires also asked about health conditions.

### Statistical analysis

Since some diabetes risk factors may be sex-specific, we conducted all analyses separately for men and women.^12^ Baseline characteristics of eligible participants were compared across the groups of SSB consumption.

We used logistic regression to assess the association between baseline SSB consumption and development of T2DM by 2013. We estimated age-adjusted odds ratios (OR) and 95% confidence intervals (CI) (model 1) and then selected variables to include in the fully-adjusted model using directed acyclic graphs (DAGs), based on the previous work with this cohort. These included age, area of residence, education, income, physical activity, consumption of fruit/vegetables, deep-fried food and alcohol, smoking, hypertension at baseline, and baseline BMI (model 2) (shown to be related to SSBs in our data^19^). We also assessed whether other variables potentially associated with an unhealthy lifestyle, (including western-style fast food intake, screen time, and other forms of sedentary behaviour) confounded that SSB-T2DM association. We found that inclusion of these factors in our models did not confound the association between SSB intake and T2DM incidence and therefore did not include these variables in our final analyses.

As it has been suggested that the relationship between SSB consumption and T2DM risk could vary by age and BMI^23^ we stratified the models by baseline BMI (<25 versus ⩾25 kg m^−2^) and age (<40 versus ⩾40 years). We also added the interaction terms of interest (SSB intake × age or SSB intake × BMI) to the main model.

We calculated population attributable fractions separately for men and women using the standard formula PAF=[(Incidence in total population−Incidence in unexposed)/Incidence in total population] × 100 to determine the proportion of T2DM in the population that could have been prevented if no-one had consumed SSBs daily. We used SSB intake at baseline as our exposure measure and calculated cumulative incidence by dividing the new cases of T2DM between 2005 and 2013 by those at risk in 2005.^24^ We then multiplied the sex-specific 8-year cumulative incidence and the PAFs from our study by the total number of men and women in the national Thai population and divided by eight to estimate the number of T2DM cases in the national Thai population that might have been prevented annually if daily SSB consumption was avoided.

#### Mediation of incident T2DM in 2013 by obesity in 2009

We conducted mediation analyses to assess the extent to which obesity in 2009 mediated the effect of SSB intake in 2005 on T2DM risk in 2013.^10, 11^ For direct comparison with previous studies we estimated the ORs and 95% CI, adjusting for covariates from the main regression model with and without BMI in 2009. To avoid mediator-outcome confounding^25^ we excluded participants who reported incident T2DM in 2009 from these analyses.

We also ran a counterfactual-based mediation analysis using Stata PARAMED,^26^ which compares two regression models: the first model regresses the outcome (T2DM incidence) on the main exposure (SSB intake), the proposed mediator (obesity) and specified covariates; the second model regresses the proposed mediator (obesity) on the exposure variable (SSB intake) and specified covariates.^25^

The mediation analysis was carried out using logistic regression since the outcome (T2DM in 2013) is binary. We dichotomized SSB intake in 2005 (main exposure) into daily intake (1+ per day) versus non-daily intake, and BMI in 2009 (our proposed mediator) into ⩾25 versus <25 kg m^2^.Covariates from the main logistic regression models were included, and an exposure mediator interaction to account for any interaction effect.^25^

**Figure 1:**
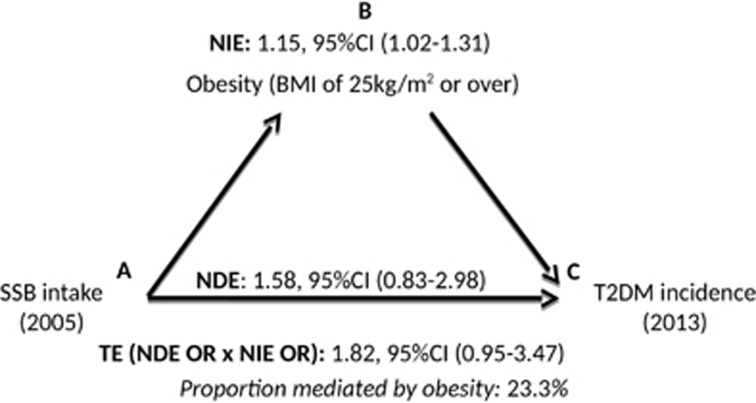
Mediation analysis investigating the association between SSB intake and T2DM incidence in 2013 mediated by obesity in 2009 in female TCS participants. Adjusted for baseline age, residence, education, income, leisure physical activity, smoking and drinking status, consumption of fruits and vegetables, consumption of deep fried food, and hypertension. NDE, natural direct effect; NIE, natural indirect effect; SSB, sugar-sweetened beverages; TE, total effect T2DM, type 2 diabetes mellitus.

We estimated the natural direct effect of SSB intake on T2DM risk and the natural indirect effect of SSB intake on T2DM risk mediated by obesity by fitting two logistic regression models; one for T2DM, conditional on SSB intake, BMI in 2009, SSB-obesity interaction, and relevant confounders; and one for obesity in 2009, conditional on SSB intake and relevant confounders. Using these two regression models we then derived the ORs for the natural direct effect of SSB intake on T2DM risk (Figures 1a–c) and the natural indirect effect mediated by obesity in 2009 (Figures 1a–c). The total effect was derived from the product of the natural direct and indirect effect. We also divided the natural logarithm of the natural indirect effect by the natural logarithm of the total effect to determine the proportion of the total association between SSB intake in 2005 and T2DM risk in 2013 mediated by obesity in 2009.

### Sensitivity analysis

It has been suggested that measures of central adiposity (including waist circumference and waist-to-height ratio) may be more informative for assessing the impact of obesity on cardio-metabolic diseases like diabetes in Asian populations.^27, 28^ Therefore, we conducted analyses to assess mediation of the SSB-T2DM association by each of: a) 2005–2009 weight gain; b) 2009 waist circumference;c) 2009 waist-to-height ratio; and d) various binary BMI cut-points (overweight (⩾23 versus <23 kg m^2^), obese I (⩾25 versus <25 kg m^2^), and obese II (⩾30 versus <30 kg m^2^)).

We also examined the association between SSB intake at baseline and the development of incident T2DM reported in 2009 to enable investigation of effects of attrition between 2009 and 2013.

All analyses were carried out using Stata (version 13.0, Australian National University, Canberra, ACT, Australia). All statistical tests were two-sided.

Ethical approval for the study was obtained from STOU Research and Development Institute (protocol 0522/10) and the Australian National University Human Research Ethics Committee (protocols 2004/344, 2009/570). All participants gave informed written consent and data were de-identified before analysis.

### Data access

Data are available through a data access agreement. All data access enquiries should be forwarded to Professor Adrian Sleigh and Associate Professor Sam-ang Seubsman (Principle investigators for the Thai Cohort Study).

### Code availability

The programming code is available from KP.

## Results

Of the 87 151 initial TCS participants, 775 did not have valid SSB data and 902 reported diabetes at baseline so were excluded. Of the remainder, 39 175 were followed-up in 2013 of whom 695 reported a new diagnosis of diabetes.

The characteristics of all TCS participants by sex and baseline SSB consumption are shown in Table 1. Men consumed SSBs more frequently than women (*P*<0.001). The median (first, third quartiles) age of participants who consumed SSBs more than daily at baseline was 28 (24, 34) among men and 25 (22, 30) among women; SSB consumption decreased with age in both sexes (*P*-trend <0.001). Frequent SSB consumption was more prevalent among those who: lived in urban areas; had lower education levels; earned a lower income; smoked; drank alcohol regularly; frequently consumed deep-fried food; consumed <two serves of fruits and vegetables per day; or exercised ⩽daily (all *P*<0.001). At baseline, men who rarely consumed SSBs were more likely to be obese (*P*<0.001).

After adjusting for confounders (Table 2, model 2), baseline SSB intake was associated with an increased odds of T2DM in 2013 among women but not men. Among women, both moderate and high SSB intakes were associated with increased odds in 2013(OR=1.6, 95% CI 1.2–2.1 and OR=2.4, 95% CI 1.5-3.9 respectively).There was no evidence that the SSB-T2DM association was modified by age or BMI in either men or women.

We estimated that ~1% of T2DM in men and ~5% in women could be attributed to daily SSB consumption. Assuming a causal SSB intake-T2DM association, ~1500 T2DM cases in men and 2700 in women per year may have been prevented in the national Thai population if daily SSB consumption was avoided.

### Mediation of incident T2DM in 2013 by obesity in 2009

Results from the logistic regression showed that amongst women, adjusting for BMI in 2009 slightly attenuated the associations between SSB consumption and development of T2DM in 2013 (unadjusted for BMI in 2009: OR=1.6, 95% CI 1.1–2.3 and OR=2.6, 95% CI 1.4–4.8 versus adjusted for BMI in 2009: OR=1.5, 95% CI 1.0-2.3 (6% attenuation) and OR=1.9, 95% CI 1.0–3.7 (27% attenuation), respectively; Supplementary Information).

In our counterfactual mediation analysis, the estimate for the natural indirect effect of SSB intake in 2005 on T2DM risk in 2013 was 1.15, 95% CI (1.02, 1.31), suggesting that 23% of the total association between 2005 SSB intake and T2DM risk in 2013 was mediated by obesity in 2009 (Figure 1).

#### Sensitivity analyses

Sensitivity analyses indicated that weight gain, waist circumference and waist-to-height ratio in 2009, as other measures of body fatness, were all mediators of the total effect of SSB intake in 2005 on T2DM risk in 2013 (Table 3). The proportions of the total effect of SSB intake on T2DM risk in 2013 mediated by these measures (2.9 to 32.9%) were similar to the proportion mediated by obesity. Using different cut-points of BMI gave mediated proportions ranging between 6.6 and 38.4%. Results in Table 3 show that for all of the investigated mediators, the proportion mediated by each of these measures increased as the cut off criteria for obesity increased.

The association between 2005 SSB consumption and risk of incident T2DM in 2009 was very similar to the association with risk of T2DM in 2013 (OR=1.6, 95% CI 1.2–2.2 and OR=2.2, 95% CI 1.3–3.6 respectively) among women suggesting that attrition between 2009 and 2013 is unlikely to have substantially influenced estimates.

## Discussion

In this prospective cohort of Thai adults we found that in women, SSB consumption was associated with increased odds of T2DM and this increased with more frequent consumption. We found that a moderate proportion of the SSB-T2DM relationship was mediated through BMI (23%) and that the proportion mediated increased with increasing BMI.

Potential limitations need to be considered when interpreting our findings. We had no information on consumption of non-carbonated sweetened beverages (that is, juices), nor did the questionnaire differentiate between sugar-sweetened and artificially sweetened beverages. The resultant misclassification is likely to have attenuated the relation between SSB intake and T2DM risk in this cohort (assuming a smaller association between artificially sweetened beverages and T2DM risk than SSBs). We also ascertained diabetes diagnoses through self-report, thus there will be some error in our classification of cases. However, a validation sub-study previously conducted amongst a sample of TCS participants indicated high accuracy of T2DM self-report, particularly among those who reported diabetes in both 2009 and 2013 (96%).^18^ Thus, misclassification of diabetes status is unlikely to have materially altered our estimates.

There is an additional study consideration that should be emphasized. SSB consumption may be a possible marker of an overall unhealthy lifestyle. Therefore, although the casual logic linking SSB intake to diabetes risk is strong, it is possible that some of the effect in our study is due to unmeasured confounding by other factors associated with an unhealthy lifestyle. We had insufficient food frequency information to estimate the contribution of SSBs to total energy intake. However, other studies found that adjusting for energy did not negate the positive association between SSB intake and risk of T2DM.^29, 30, 31^ Loss to follow-up was substantial with ~50% of the baseline cohort retained after eight years. For most variables, baseline distributions did not vary between participants who remained in the study and those not followed-up after 8 years. For some variables (regular SSB consumers, younger participants, and those underweight), rates of attrition were slightly higher. Given the evidence that these variables influence the risk of diabetes in this cohort, the higher attrition may have altered the SSB-T2DM effect estimation. However, the SSB-T2DM associations observed using only the 2009 incidence data (70% of baseline cohort) were similar suggesting such bias is likely to be minimal.

Our finding of an association between consumption of SSBs and increased risk of T2DM in women is consistent with findings from most studies in African,^10^ Caucasian^10, 11, 32, 33^ and Asian populations.^12^ One previous study found no association between SSB consumption and T2DM risk for men or women, although age differences may explain this; SSB consumption is more common in younger adults,^7^ and the mean age of the Atherosclerosis Risk in Communities Study participants at baseline was 53.6^(ref. 23)^ compared to 30.5 in our cohort.

A partial explanation for the sex specificity of the association may relate to energy requirements. Women generally have lower muscle mass than men hence lower metabolic energy needs^34^ so similar SSB intake would contribute a larger proportion of total energy intake.^12^ It may be that an association in men is only apparent at higher consumption levels than we observed here. Some studies found a relationship only in non-obese individuals.^10, 11, 12, 32, 33^ We did not find effect-modification by obesity in this population. However, the prevalence of daily SSB consumption and obesity among these women was low and we may have lacked the statistical power to detect effect-modification by obesity in this cohort.

In keeping with previous studies, our results suggest that a moderate proportion of the SSB-T2DM relationship was mediated through BMI.^14, 31, 33^ The proportion mediated through BMI increased with increasing obesity cutoffs, possibly reflecting the increasing T2DM risk with increasing BMI^22, 35^ or it may be that more obese participants were regularly drinking larger amounts of SSB. Most studies have investigated mediation by adjusting for BMI (the mediator) and assessing the change in the magnitude of the association. This approach can produce bias due to unmeasured mediator-outcome confounding or interaction between the exposure and mediator (SSB intake and BMI).^36^ Here we assessed mediation using both a counter-factual mediation analysis and by adjusting for BMI in a standard regression model. Results were very similar using both approaches suggesting that unmeasured mediator-outcome confounding or interaction between the exposure and mediator are minimal for this association.

We had expected that a large proportion of the association between SSB intake and T2DM would be mediated by weight gain or obesity because SSBs can stimulate intake of other high glycaemic foods^37, 38^ leading to higher total caloric intake.^37, 39, 40^ However, regular SSB consumption may increase T2DM risk through mechanisms independent of weight gain or obesity. For instance, high glycaemic loads from SSBs lead to repeated high insulin demand, which can contribute to compromised beta (β) cell function.^38^ This may be particularly problematic in low and middle-income country Asian adults who may have experienced intrauterine or early childhood under-nutrition. This can lead to the under-development of β cell mass and an increased risk of T2DM later in life^41^ independent of weight gain, especially with exposure to energy-dense foods like SSBs.^42, 43^

## Conclusion

The findings from this cohort suggest that at this point of the Thai health-risk transition SSB intake is increasing the risk of T2DM in women. As SSBs have no nutritional value and do not protect against disease they are an ideal target for public health efforts aimed at preventing increasing national T2DM incidence. Reducing the incidence and prevalence of T2DM in Thailand will require a multi-faceted approach. Targeting SSBs could serve as one focal point to prevent a national rise in the incidence of T2DM.

## Figures and Tables

**Table 1:** Baseline SSB consumption by sociodemographic and behavioural characteristics of eligible participants in the Thai Cohort Study^a^

*Characteristics*	*SSB consumption* N=*85* *474*^a^			P-*value*^b^
	*Rarely (*N=*44* *784)* n *(%)*^c^	*1*–*6 per week (*N=*34* *113)* n *(%)*	⩾*1 a day* (n=*6577*) n *(%)*	
*Men at risk in 2005*	17 805 (46)	17 657 (46)	3013 (8.0)	<0.001^d^
Median age (1st, 3rd quartile)	33 (27–40)	29 (25–35)	28 (24–34)	<0.001^c^
Obese (⩾25.00 kg m^−2^)	4197 (24)	3616 (21)	621 (21)	<0.001
>High school qualification	8179 (46)	7863 (45)	1183 (39)	<0.001
Urban residence	8422 (48)	8918 (51)	1832 (61)	<0.001
Income ⩾10,001 baht per month	7924 (46)	6527 (38)	1026 (35)	<0.001
Regular/social drinkers	13 414 (76)	14 106 (81)	2261 (76)	<0.001
Current smokers	3240 (19)	3857 (23)	788 (28)	<0.001
⩾2 serves fruits/veg per day	16 425 (96)	16 533 (97)	2695 (95)	<0.001
Deep-fried food eaten ⩾1 per day	1917 (11)	2870 (16)	1148 (38)	<0.001
Physical activity (⩾8 per week)	11 073 (68)	11 452 (70)	1847 (67)	<0.001
				
*Women at risk in 2005*	26 977 (57)	16 455 (35)	3564 (8.0)	<0.001^d^
Median age (1st, 3rd quartile)	28 (24–35)	26 (23–31)	25 (22–30)	<0.001^c^
Obese (⩾25.00 kg m^−2^)	2613 (10)	1582 (10)	382 (11)	0.108
>High school qualification	15 390 (57)	9325 (57)	1761 (50)	<0.001
Urban residence	13 991 (52)	8601 (53)	2100 (59)	<0.001
Income ⩾10,001 baht per month	8246 (31)	4143 (26)	814 (23)	<0.001
Regular/social drinkers	13 268 (50)	9422 (58)	2069 (59)	<0.001
Current smokers	212 (1.0)	171 (1.0)	83 (2.0)	<0.001
⩾2 serves fruits/veg per day	25 696 (98)	15 557 (98)	3275 (97)	<0.001
Deep-fried food eaten ⩾1 per day	3153 (12)	2754 (17)	1225 (34)	<0.001
Physical activity (⩾8 per week)	12 643 (51)	7198 (47)	1486 (45)	<0.001

Abbreviation: SSB, sugar-sweetened beverages.

a Eligible participants at baseline did not have T2DM (*n*=902) and were not missing SSB data (*n*=775). Note for each tabulated characteristic the numbers vary a little due to missing data.

b *χ*^2^ comparing baseline characteristics among participants by SSB consumption.

c number (%) in each category. For median age numbers in brackets are first and third quartiles and the final column records p-trend.

d *χ*^2^ comparing SSB consumption by sex.

**Table 2:** Associations between SSB intake in 2005 and incidence of T2DM between 2005 and 2013 by sex

*SSB intake at baseline in 2005*	*Odds ratios (ORs) and 95% Confidence intervals (CI)*		
	*Cases by 2013/at risk in 2005*	*Model 1 OR (95% CI)*	*Model 2 OR (95% CI)*
*Men*			
Rarely	236/8860	1	1
1–6 times per wk	168/7516	1.1 (0.9–1.3)	1.0 (0.8–1.2)
⩾1 per day	33/1083	1.6 (1.1–2.3)	1.3 (0.9–2.1)
*P* trend		0.04	0.55
			
*Women*			
Rarely	142/13 291	1	1
1–6 times per wk	88/7133	1.5 (1.1–2.0)	1.6 (1.2–2.1)
⩾1 per day	28/1292	2.8 (1.8–4.2)	2.4 (1.5–3.9)
*P* trend		<0.001	<0.001

Abbreviations: BMI, body mass index; wk, week.

Model 1: Age adjusted.

Model 2: Adjusted for age, residence, education, income, physical activity, smoking and drinking status, consumption of fruits and vegetables, consumption of deep fried food, hypertension at baseline, and baseline BMI.

**Table 3:** Mediation analysis investigating the association between SSB intake and T2DM incidence in 2013 mediated by various measures of adiposity in 2009 in female TCS participants

*Mediator in 2009*	*Natural direct effect* *OR (95% CI)*	*Natural indirect effect* *OR (95% CI)*	*Total effect* *OR (95% CI)*	*Proportion mediated %*
*Body mass index (BMI/m*^*2*^)				
BMI–overweight (23 kg m^−2^)	1.74 (0.93–3.26)	1.04 (0.95–1.15)	1.81 (0.96–3.42)	6.6
BMI–obese I (25 kg m^−2^)	1.58 (0.83–2.98)	1.15 (1.02–1.31)	1.82 (0.95–3.47)	23.3
BMI–obese II (30 kg m^−2^)	1.50 (0.77–2.93)	1.29 (1.04–1.61)	1.94 (0.98–3.84)	38.4
				
*Weight gain (2005-2009)*				
Gained 5 kg or more	1.95 (1.05–3.61)	1.02 (0.97–1.08)	1.99 (1.07–3.69)	2.9
Gained 10 kg or more	1.91 (1.03–3.56)	1.03 (0.96–1.11)	1.98 (1.06–3.67)	4.3
				
*Waist Circumference*				
80 centimetres or over	1.62 (0.82–3.21)	1.07 (0.98–1.18)	1.74 (0.88–3.46)	12.2
85 centimetres or over	1.43 (0.84–4.06)	1.19 (1.03–1.38)	1.71 (0.85–3.44)	32.4
				
*Waist-to-height ratio*				
0.5 or over	1.43 (0.70–2.93)	1.09 (0.97–1.21)	1.56 (0.76–3.20)	19.4
0.6 or over	1.34 (0.63–2.88)	1.16 (0.96–1.42)	1.57 (0.76–3.27)	32.9

Adjusted for baseline age, residence, education, income, leisure physical activity, smoking and drinking status, consumption of fruits and vegetables, consumption of deep fried food, and hypertension.

*Proportion mediation=log(OR^NIE^)/log(OR^TE^) × 100% where NIE represents the natural indirect effect and TE represents the total effect.
