# Supplementary material for: Consumption of sugar-sweetened beverages and type 2 diabetes incidence in Thai adults: results from an 8-year prospective study
Source: Nutr Diabetes. 2017 Jun 19;7(6):e283–. doi: 10.1038/nutd.2017.27 (PMC5519187; doi:10.1038/nutd.2017.27)
Supplement: Supplementary Information [file nutd201727x1.docx]

| **Supplement 1**: Associations between SSB intake in 2005 and incidence of T2DM in 2013 by sex | | | | | | |
| --- | --- | --- | --- | --- | --- | --- |
|  | | Odds Ratios (ORs) and 95% Confidence Intervals (CI) | | | | |
| SSB intake at baseline in 2005 | Cases in 2013/  At risk in 2005 | | Model 1  OR(95% CI) | Model 2  OR(95% CI) | Model 3  OR(95% CI) |  |
| Men |  | |  |  |  |  |
| Rarely | 138/8,762 | | 1 | 1 |  |  |
| 1-6 times/wk | 98/7,446 | | 1.1(0.8-1.4) | 1.0(0.7-1.3) | 0.9(0.7-1.3) |  |
| >1 per day | 18/1,068 | | 1.4(0.9-2.4) | 1.0(0.6-1.9) | 1.1(0.6-2.0) |  |
| P trend |  | | 0.24 | 0.95 | 0.85 |  |
|  |  | |  |  |  |  |
| Women |  | |  |  |  |  |
| Rarely | 88/13,237 | | 1 | 1 |  |  |
| 1-6 times/wk | 50/7,095 | | 1.3(0.9-1.9) | 1.6(1.1-2.3) | 1.5(1.0-2.3) |  |
| >1 per day | 16/1,280 | | 2.4(1.4-4.2) | 2.6(1.4-4.8) | 1.9(1.0-3.7) |  |
| P trend |  | | <0.01 | <0.01 | 0.01 |  |

Model 1-Age adjusted

Model 2-Adjusted for age, residence, education, income, physical activity, smoking and drinking status, consumption of fruits and vegetables, consumption of deep fried food, hypertension at baseline

Model 3-Adjusted for age, residence, education, income, physical activity, smoking and drinking status, consumption of fruits and vegetables, consumption of deep fried food, hypertension at baseline, and BMI in 2009
